# Design and evaluation of AE4W: An active and flexible shaft-driven shoulder exoskeleton for workers

**DOI:** 10.1017/wtc.2024.19

**Published:** 2025-02-25

**Authors:** Marco Rossini, Sander De Bock, Vincent Ducastel, Gabriël Van De Velde, Kevin De Pauw, Tom Verstraten, Dirk Lefeber, Joost Geeroms, Carlos Rodriguez-Guerrero

**Affiliations:** 1 Brussels Human Robotics Research Center (BruBotics), Vrije Universiteit Brussel, Brussel, Belgium; 2 Robotics & Multibody Mechanics Research Group (R&MM), Vrije Universiteit Brussel, Brussel, Belgium; 3 Human Physiology and Sports Physiotherapy Research Group, Vrije Universiteit Brussel, Brussel, Belgium; 4 Flanders Make, Lommel, Belgium; 5 IMEC, Leuven, Belgium; 6 Department of Mechanical Engineering, KU Leuven, Leuven, Belgium

**Keywords:** active shoulder exoskeleton, occupational exoskeleton, flexible shaft-driven remote-actuation

## Abstract

The wide adoption of occupational shoulder exoskeletons in industrial settings remains limited. Passive exoskeletons were proved effective in a limited amount of application scenarios, such as (quasi-)static overhead handling tasks. Quasi-active devices, albeit representing an improved version of their passive predecessors, do not allow full modulation of the amount of assistance delivered to the user, lacking versatility and adaptability in assisting various dynamic tasks. Active occupational shoulder exoskeletons could overcome these limitations by controlling the shape of the delivered torque profile according to the task they aim to assist. However, most existing active devices lack compactness and wearability. This prevents their implementation in working environments. In this work, we present a new active shoulder exoskeleton, named Active Exo4Work (AE4W). It features a new flexible shaft-driven remote actuation unit that allows the positioning of the motors close to the wearer’s center of mass while it maintains a kinematic structure that is compatible with the biological motion of the shoulder joint. in vitro and in vivo experiments have been conducted to investigate the performance of AE4W. Experimental results show that the exoskeleton is kinematically compatible with the user’s workspace since it does not constrain the natural range of motion of the shoulder joint. Moreover, this device can effectively provide different types of assistance while the user executes various dynamic tasks, without altering perceived comfort.

## 1. Introduction

Work-related musculoskeletal disorders (WRMD) at the level of the shoulder affect more than 50% of the European working population (Govaerts et al., 2021). The probability of suffering from WRMD increases with age, to such an extent that it affects almost 70% of workers above the age of 55 (EUOSHA, 2020). Currently, production and productivity losses related to WRMD have been estimated to amount to almost 3% of the European GDP (Bevan, 2015). Given the projected aging of the workforce (Mullan et al., 2017; Rudnicka et al., 2020), managing WRMD is clearly one of the primary occupational, societal, and economic challenges for the future (Menegon and Fischer, 2012).

Despite this emerging challenge, there is little evidence supporting the effectiveness of conservative ergonomic interventions (e.g., job rotation) for preventing shoulder-related WRMD (Verhagen et al., 2013). However, new wearable technologies, such as occupational exoskeletons can be a potential preventive measure (De Looze et al., 2016; NIOSH, 2019), as they can relieve the human musculoskeletal system from non-ergonomic workload-induced stresses (Moeller et al., 2022).

In the past few years, many companies and research institutions have been showing a growing interest in occupational shoulder exoskeletons (De Bock et al., 2022a). However, large-scale implementation of this technology remains limited, and often only tested in a constrained environment (Crea et al., 2021; Howard et al., 2020).

The majority of the commercially available shoulder exoskeletons are passive devices (de Vries et al., 2019; Hyun et al., 2019; Maurice et al., 2019; Pacifico et al., 2020; Van Engelhoven et al., 2019), conceived to assist the workers during the executions of overhead handling tasks. They do not require an external source of power to assist, as they use gravity compensation mechanisms to deliver support at the level of the shoulder joint. The type of support is generally an assistive torque, whose magnitude and shape are fixed by design, aimed at compensating for a portion of the user’s arm weight during (quasi-)static overhead working postures. As a result, passive exoskeletons tend to perform very well in laboratory tests, where the ideal application scenario they are designed for is reproduced (De Bock et al., 2023; Moeller et al., 2022; van der Have et al., 2021). On the other hand, they underperform in real-life situations (De Bock et al., 2020; De Vries and De Looze, 2019) where various highly dynamic tasks occur.

The effectiveness of an exoskeleton is correlated to the assistance it can deliver (de Vries et al., 2019). The direction and magnitude of the assistive torque provided by passive exoskeletons are fixed by the design of the exoskeleton structure and its spring-based actuation mechanism. As a result, only one assistive strategy can be adopted, normally consisting of the delivery of torques that support arm elevation, meaning that the exoskeleton assistance can also be perceived as resistance when the user moves in the opposite direction (Ramella et al., 2024).

To find a trade-off between assistance and resistance, passive exoskeletons usually do not compensate for more than about 50% of the gravitational load of the arm.

Recently, quasi-active shoulder exoskeletons (“Fraco, Mawashi, Quebec, Canada,”, 2020; Grazi et al., 2020, 2022; Otten et al., 2018) have been developed to overcome the limitations of a passive exoskeleton. Similar to passive exoskeletons, they implement gravity compensation mechanisms with active modulation of the amount of support delivered. By doing so, they decouple the amount of assistance delivered from the amount of resistance opposed to the user. However, the shape, sign, and peak assistance of the implemented torque profile are still dictated by the exoskeleton’s gravity compensation mechanism. Therefore, in line with their passive versions, these exoskeletons are still limited to the specific application scenario they are designed for.

Overcoming the limitations of the passive and quasi-active devices requires active wearable robots. Most active exoskeletons for the upper limb have been conceived as rehabilitation platforms (Gopura et al., 2011). As a result, such devices are not suited for realistic, and thus dynamic, working environments since they lack portability and high-bandwidth interaction control. To the best of our knowledge, four active and portable devices have been currently developed to support the shoulder during the execution of occupational tasks: the Stuttgart Exo-Jacket (Ebrahimi, 2017), the Shoulder-SideWINDER (Park et al., 2021), the commercially available S700 exoskeleton (exoIQ, Hamburg, Germany) (Sänger et al., 2022), and a customized version of the passive EVO shoulder exoskeleton (Ekso Bionics Holdings Inc, CA) (Nasr et al., 2023a; Nasr et al., 2023b).

The Stuttgart Exo-Jacket is a robotic device equipped with 12 degrees of freedom (DOF), three of which are actively actuated. Two motors, situated at the shoulder level, drive the flexion-extension and ab-/adduction movements of the human joint, while a third motor is dedicated to the elbow joint. Despite its capacity to generate up to 40 Nm of torque around the shoulder joint, this exoskeleton’s application remains confined to research environments.

The Shoulder-SideWINDER is an 8-DOF exoskeleton designed to actively support the shoulder joint. Its self-aligning mechanism mitigates misalignment between the user’s shoulder joints of the user and the exoskeleton. As it remains in the developmental phase, comprehensive data regarding the performance of its cable-driven actuation system are currently unavailable.

The S700 exoskeleton provides active shoulder support for tasks, driven pneumatically by a battery-powered compressor. The device features different assistive presets adjustable via a mobile application, but the exact assistive characteristics of this commercially available device are not public. In addition, pneumatic actuators are limited in motion control capabilities and have limited feedback mechanisms, compromising the potential to precisely and accurately assist the user.

The active EVO exoskeleton comprises a motor at the exoskeleton’s shoulder joint and utilizes a human-in-the-loop approach to control the exoskeleton (Nasr et al., 2023c), and experiments remain limited to a seated lifting task in the sagittal plane (Nasr et al., 2023b; Nasr et al., 2023c).

Several technological barriers can prevent wide adoption of active exoskeletons (Babič et al., 2021). First, the complexity of the upper limb, especially the shoulder complex, makes the design of a wearable robot difficult (Gopura et al., 2016). There is no final solution to achieve kinematic compatibility between the human structure and the exoskeleton structure (Näf et al., 2018. To solve this problem, complex/bulky designs are usually proposed, albeit unfeasible to implement in portable structures (Blanco et al., 2022; Sylla et al., 2014). Lighter designs use misalignment compensation mechanisms (Näf et al., 2018 and self-alignment mechanisms. An example of the latter typology has been recently implemented in (Park et al., 2021) to reduce the misalignment between the actuated joint of the robot and the user’s shoulder joint. However, this solution has proven efficacy only for specific users with specific anthropometric measures.

Kinematic incompatibility not only restricts the anatomical range of motion (ROM) in human joints (Schiele, 2009), but also leads to user discomfort. Moreover, it can give rise to forces and torques at the physical human–robot interface, potentially posing harm to the user (K. P. Langlois, 2022). Therefore, the exoskeleton structure should not only retain the mobility of the shoulder joint but also preserve its natural ROM. This is challenging to achieve while keeping a minimalist exoskeleton structure with a small footprint.

The actuation system should be responsive and backdrivable (Toxiri et al., 2019); the exoskeleton’s mass distribution should be as such to relocate some of the components as close as possible to the user center of mass so as to minimize the inertia of the exoskeleton’s distal components, reduce power consumption (Rodriguez-Cianca et al., 2019), improve comfort and balance.

**Figure 1.**
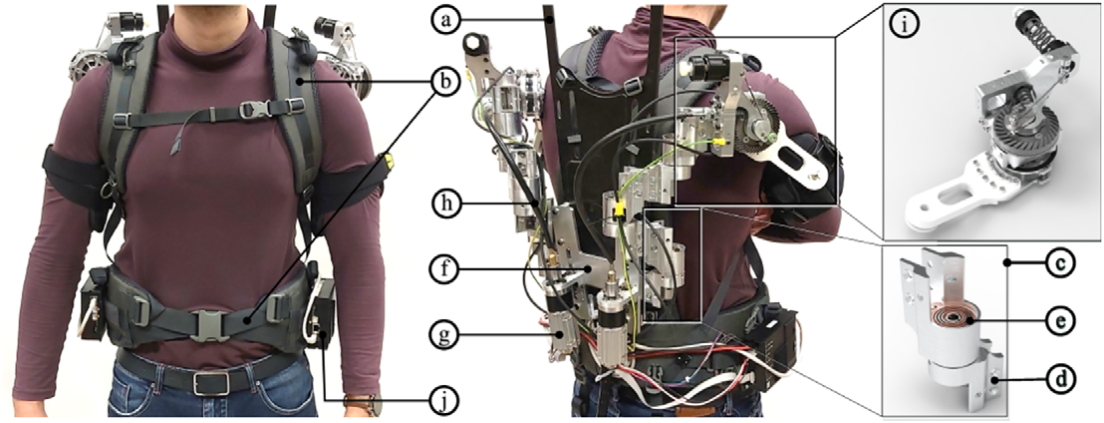
AE4W prototype: (a) back plate; (b) shoulder and hip interfaces; (c) custom-made hinge; (d) connection link; (e) torsional spring; (f) adjustable clamp; (g) actuators; (h) flexible shaft; (i) End-Effector Module (EEM) of the RAS; and (j) EPOS4 controller. It is worth noting that the chosen design features aim at minimizing the frontal footprint of the exoskeleton that does not protrude from the body of the user. This might improve the usability of the device in an industrial scenario.

In this work, a new powered shoulder exoskeleton, Active Exo4Work (AE4W), is presented (Figure 1). The AE4W structure is kinematically compatible with the shoulder joint resulting from a structural study conducted in previous work (Rossini et al., 2021c) and further detailed in the next section. A new backdrivable remote actuation system (RAS) has been integrated into the exoskeleton structure. Said actuation system comprises a flexible shaft, as in (Rodriguez-Cianca et al., 2019), adapted to transmit and measure a torque. A further custom-made torque sensor has been integrated distally into the RAS to allow redundancy of force data acquisition, robustness, and safe human–robot interaction (Pedrocchi et al., 2013). The off-joint actuation system has also enabled the redistribution of the exoskeleton components, seeking a smaller exoskeleton frontal footprint. The RAS has been controlled in torque and its design has been validated on a test bench. Finally, a preliminary pilot study including seven participants has been conducted to measure the performance of AE4W in terms of compatibility of its kinematic structure and to validate the efficacy of the torque controller with real-life human motions.

## 2. Materials and methods

### 2.1. AE4W kinematic synthesis and realization

The structural synthesis of a wearable robot is a problem with numerous potential solutions (Ye and Li, 2019). Therefore, identifying the right kinematically compatible structure poses a challenging task. In reality, the eventual design decision is often driven by the engineer’s insight and expertise. Reducing the dimension of the design space to a number of solutions that can be easily analyzed by the designer, can lead to a better choice.

In our previous studies (van der Have et al., 2021; Rossini 2021; Rossini 2022; Rossini et al., 2021c), a design support system was developed for the automatic enumeration of spatial exoskeleton kinematic chains compatible with the anatomy of the human shoulder. The following requirements were considered before the exoskeleton kinematic synthesis:

- The exoskeleton is considered a parallel kinematic chain connected to the human kinematic chain through two anchor points positioned on the upper arm and the back;
- The mobility of the whole parallel structure constituted by the exoskeleton and the human arm should allow the anatomical mobility of the shoulder complex.
- The presence of a misalignment compensation mechanism (MCM) constituted by a triplet of rotational joints in series (the RRR-MCM (Näf et al., 2018), has been sought in a proximal position in the exoskeleton kinematic structure;
- Non-redundant kinematic structures with isolated singularities are allowed (Gogu, 2002);
- The connections between the links of the exoskeleton have been realized with a combination of prismatic joints, revolute joints, spherical joints, and universal joints.

**Figure 2.**
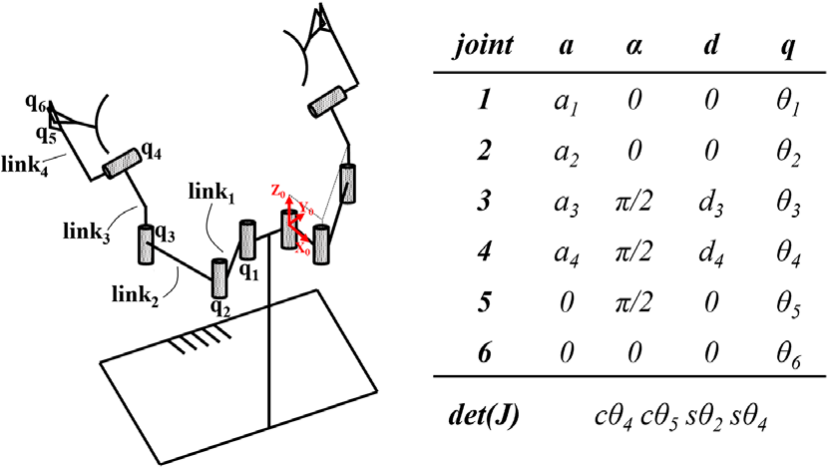
Denavit-Hartemberg parameters and determinant of the Jacobian 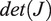 are reported for the AE4W kinematic structure. The arbitrary choice for the origin reference frames is highlighted in red.

Six kinematic chains with equal mobility and isolated singularities were found (van der Have et al., 2021; Rossini et al., 2021c). Only one of them (Figure 2) presented some characteristics that are useful both for the improvement of the ergonomics of the exoskeleton and the RAS implementation. First, it presents an axial offset 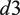 , that allows the designer to freely choose the position of the exoskeleton anchor point on the back of the user. Moreover, parameter 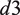 releases the constraint for the exoskeleton joints 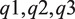 , to be co-planar, thus avoiding collision problems between their interconnecting links. In addition, offset 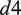 can be implemented to control the relative distance between between joint 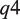 and joint 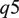 . These two joints represent respectively the last joint of the exoskeleton structure (after which the physical human–robot interface with the arm takes place) and the shoulder joint of the exoskeleton. Consequently, 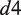 can be chosen as such to keep 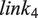 close to the arm of the user, thus limiting the lateral footprint of the exoskeleton. Finally, the second kinematic structure has an orthogonal offset 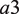 , needed for the realization of the RAS, as described in the next section.

The exoskeleton structure (Figure 1) is mounted onto a back plate (a – KIUI, Dixon, CA) made of hi-modulus carbon fiber. The back plate is connected with the hip belt and the shoulder harnesses (b – KIUI, Dixon, CA), which are equipped with adjustable clasps and buckles for size adjustment.

Three parallel custom-made rotational joints (RRR-MCM) in aluminum (c), originate from the back plate of the exoskeleton. The way they are implemented does not limit their ROM, so they can assume a folded configuration without the risk of collision between their connecting links (d). Additionally, joints 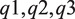 are spring-loaded. The equilibrium position of the spring (SPF-0912, Durovis, Switzerland; e) is set through a set screw in a way that the torque increases approaching the singular configuration of the exoskeleton, thus passively implementing an escapement strategy.

The anchor point of the RRR-MCM is realized through an adjustable clamp (f) that allows the whole exoskeleton structure to be translated up and down relatively to the torso of the user to fit different body types.

The actuators (g), mounted on the exoskeleton back plate, are decoupled from the driven joints thanks to a pair of flexible shafts (h). The latter ones transfer torque to the End-Effector Module (EEM) (i) so forming the RAS, further detailed in the next section.

The length of the links has been optimized with a heuristic approach to maximize the user’s freedom of motion (Huysamen et al., 2020) whilst being as short as possible. The complete list of the exoskeleton parameters can be found in Table 1. The final weight of the prototype is ~10 kg comprising 3.7 kg for the back plate and the actuators mounted on it and 3.2 kg for each arm of the exoskeleton.

**Table 1.** Denavit–Hartenberg parameters of the AE4W

a [mm]	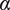	d [mm]	Joint variable, ROM
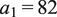	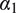 =0	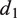 =0	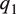 , ( 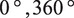 ), passive
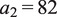	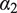 =0	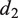 =0	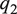 , ( 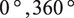 ), passive
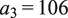	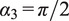	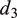 = − 19	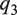 , ( 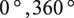 ), passive
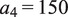	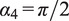	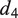 = − 19	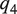 , (−30°, +180°), active
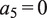	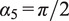	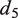 =0	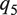 , ( 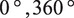 ), passive
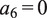	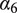 =0	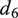 =0	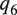 , ( 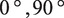 ), passive

### 2.2. Remote actuation system implementation

**Figure 3.**
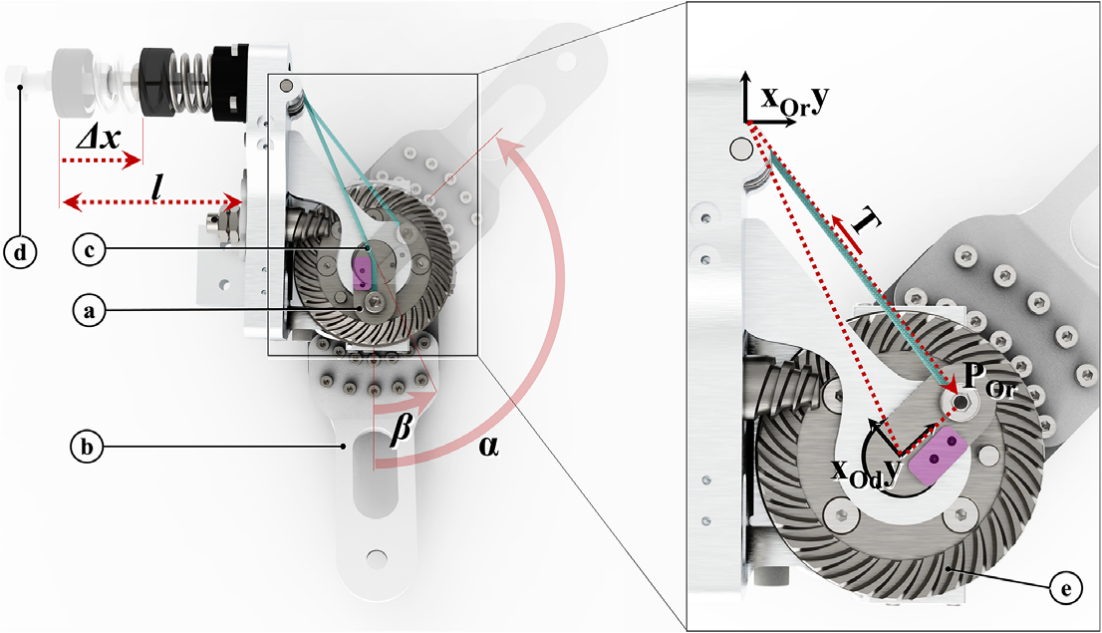
PEM: (a) cam profile; (b) exoskeleton link; (c) Dyneema cable (blue); (d) pretension screw; and (e) hypoid gearbox. For angle 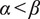 the cable straight path is deviated by the edge highlighted in pink.

The power requirements listed by (Huysamen et al., 2020), have been considered before the drive unit selection. The off-the-shelf flexible shafts have been chosen for their bending compliance; they have been cut to the right length before the execution of the tests. Finally, the EEM has been designed to fulfill a threefold function. First, it implements a parallel-elastic torque generation mechanism (PEM, Figure 3), conceived as an add-on module of the RAS to boost its performance (Verstraten et al., 2016) and increase its safety, assuring a passive functioning of the RAS even in case of a power outage. Then, a second stage of reduction (Figure 3, e), implemented with a custom-made backdrivable hypoid gearbox (gear ratio 15:1), shifts the axis actuated by the flexible shaft of 90°, making it coincide with the shoulder joint of the exoskeleton. Finally, a custom-made Output Torque Sensor (OTS) has been integrated into the EEM design to allow redundancy of force data acquisition for a safer human–robot interaction (Pedrocchi et al., 2013).

The RAS (Figure 1) is composed of three different modules: the drive unit constituted by a DC motor (EC-4pole, 200 W, maxon Group, Switzerland) equipped with a backdrivable 3.5:1 planetary gearbox (GP42, maxon Group, Switzerland) and a magnetic encoder (HEDL 5540, 500 counts per revolution, maxon Group, Switzerland); a flexible shaft (Dremel 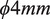 , USA); the EEM.

The drive unit is placed in a metabolically advantageous position, that is, close to the center of mass of the user, to reduce the inertia of the system at the level of the actuated distal joint (Rodriguez-Cianca et al., 2019). The flexible shaft connects the motor with the driven joint. Although the spatial configuration of the latter varies with the kinematic configuration of the user’s arm, the flexible shaft allows a continuous power transmission thanks to its bending flexibility. Moreover, the compliance properties of the shaft can enable the RAS to have characteristics proper of series elastic actuators, improving the safety of the interaction with humans and paving the way to the implementation of indirect torque control strategies.

#### 2.2.1. Parallel-elastic torque generation mechanism

The PEM has a twofold function: (i) boost the RAS performance in terms of the amount of delivered torque to the user; (ii) assure a passive functioning of the AE4W. The latter aspect is important for safety reasons, preventing an abrupt fall in torque in case of a power outage or interruption of the motor operation. The PEM function is to generate a torque profile that fulfills the following list of requirements:

- Maximum delivered torque 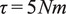 ; This amount of torque equals the peak of assistance of the majority of the passive shoulder exoskeleton on the market and it has been proven to effectively reduce shoulder muscle activity (de Vries et al., 2019; Hyun et al., 2019; Maurice et al., 2019; Pacifico et al., 2020; Van Engelhoven et al., 2019);
- Peak of assistance occurring between 90° and 130°;
- Low support for shoulder elevation angles 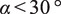 , which do not require assistance (van der Have et al., 2021).

The PEM design (Figure 3) is based on a previously developed mechanism (van der Have et al., 2021) that makes use of a wrapping cam (a) to convert the spring (Sodemann 13400, Denmark) elastic force into a torque. The cam rotates with the exoskeleton link (b) and it is attached to a cable (Dyneema 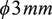 , The Netherlands, c). The latter connects point 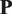 (on the cam) with the free end of the spring so that a rotation 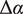 of the exoskeleton link corresponds to a compression 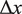 of the spring and a value of tension 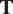 on the cable. It is worth noting that for angles 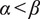 , the cable wraps around the cam’s edge, located in the coincidence of the center of rotation of the cam (origin of the reference frame 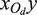 ). In this configuration, the moment arm of the cable tension 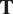 is nearly zero, as well as the magnitude of the resulting torque generated by the PEM.

The position of the cable attachment point 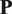 in the 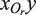 reference frame can be described as a function of 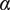 :
(2.1)

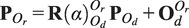

where 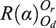 represents the rotational matrix describing the orientation of the reference frame 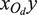 relative to the reference frame 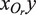 and 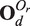 is the vector that describes the position of 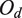 within the reference frame 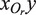 .

Consequently, the compression of the spring 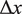 , can be computed as:
(2.2)

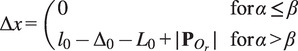

where 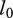 is the unloaded length of the spring, 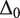 is the pre-compression of the spring adjusted with the pretension screw and 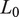 is the length of the portion of the cable running between 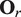 and 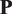 for 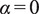 .

Finally, the moment of the cable tension 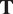 , calculated with respect to the point 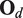 can be expressed as:
(2.3)

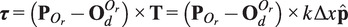

 where 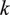 represents the stiffness coefficient of the employed spring and 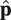 is the versor indicating the directional components of the tension 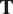 .

Given the model, the values of the described parameters are chosen to meet the requirements reported above for the assistive profile the PEM should provide. The complete list of parameters is reported in Table 2.

**Table 2.** PEM parameters

Parameter	Value
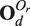	[42–82 0] mm
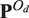	[20 0 0] mm
K	8.2 N/mm
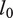	94.2 mm
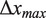	20 mm

**Figure 4.**
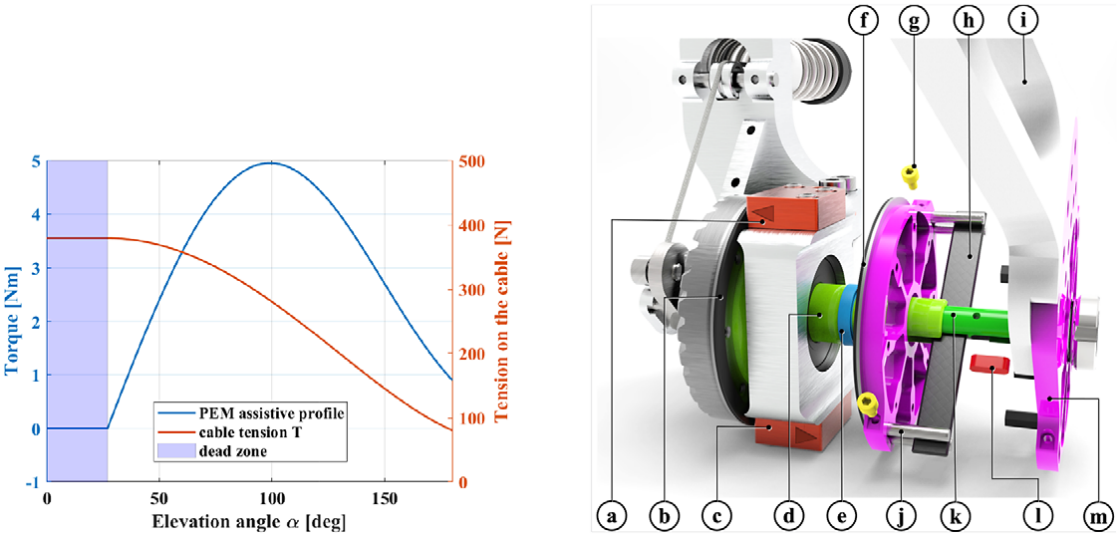
*Left*: Torque profile generated by the PEM for 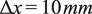 . The corresponding cable tension is reported on the right *y*-axis. Despite the high tension on the cable in the low-assistance area (shaded in light blue), the PEM output torque is zero. *Right*: OTS components: (a) encoder of the hypoid axis – reader; (b) encoder of the hypoid axis – magnetic ring; (c) encoder of the exoskeleton link – reader; (d) output shaft of the hypoid gearbox; (e) ball bearing; (f) encoder of the exoskeleton link – magnetic ring; (g) fasteners for the carbon fiber beam’s clamping pins; (h) carbon fiber beam; (i) exoskeleton link; (j) clamping cylindrical pins; (k) clamping insert; (l) feather key; and (m) radial component for the anchoring of the exoskeleton link.

The choice of the PEM parameters results in the torque profile shown in Figure 4.

#### 2.2.2. Output torque sensor design

The OTS (Figure 4) is embedded in the RAS and constitutes the anchor point for the exoskeleton link (i).

Its sensitive element is constituted by a carbon fibre beam (h), which transfers the power in output from the hypoid gearbox to the exoskeleton link. The rigid connection between the gearbox output axis (d) and the beam, is realized through a steel insert (k), and a key (l). The relative rotation between the gearbox axis and the radial components (m) is allowed thanks to two bearings (e). Clamping the beam’s free ends (j) to the radial components (m) enables the power transfer toward the exoskeleton link. The angular position of the hypoid gearbox shaft and the exoskeleton link are respectively gauged by two incremental magnetic encoders (LM10, RLS, Ljubljana, Slovenia – a, c), whose differential measurement represents the carbon fiber beam deflection. The latter parameter can be related to the applied load on the beam, giving an estimation of the output torque generated at the output of the RAS. It is worth noting that the angular resolution of the encoders (7808 counts per revolution, that is, 0.05°) determines the lower bound for the resolution of the torque estimation. Therefore, the establishment of the requirements for the torque sensor can guide the selection of the aspect ratio of the beam.

Torque sensors for human–robot interaction do not have stringent requirements in terms of stiffness and resolution, unlike commercial sensors usually used in robotics (Lorenz et al., 1999). Consequently, we aim to realize a sensor with a theoretical resolution, whose order of magnitude is 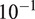 Nm. Given the encoders we employed, the smallest displacement we can measure is 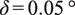 . Once the required resolution for the torque sensor has been set, the moment of inertia 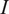 of the beam section can be chosen by assuming that its deflection follows a cantilever beam model:
(2.4)

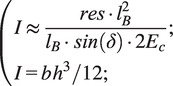

 where 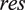 represents the target resolution; 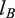 and 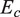 are, respectively, the free length of the beam and the carbon fiber beam Young’s modulus. Since 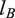 results defined by the design shown in Figure 4 and 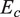 is a property of the material which the beam is made of, Eqn. 2.4 results verified for the beam cross-section 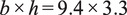 mm^2^ (Goodwinds Composites, USA).

#### 2.2.3. Remote actuation system control architecture

**Figure 5.**
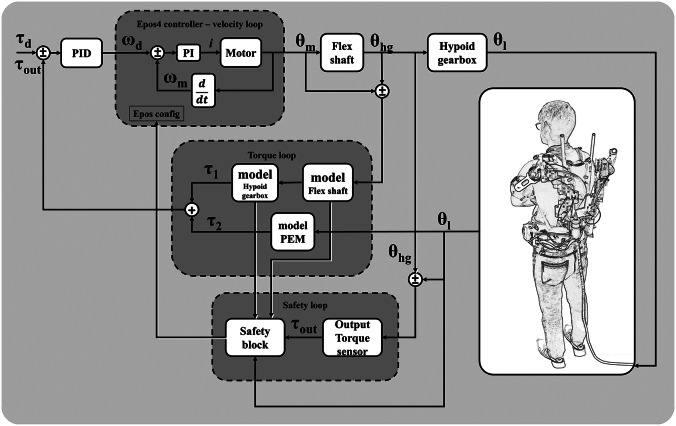
RAS control architecture. Three control loops are implemented to control the interaction torque with the user safely. An indirect torque controller exploits the shaft, hypoid gearbox, and PEM models to estimate the output torque 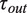 applied on the user. In cascade, a velocity control loop regulates the motor position 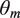 . A safety loop running in parallel to the torque control loop estimates 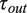 on the base of the differential measurement between the output link 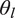 and the hypoid gear 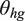 .

The RAS control scheme (Figure 5) is composed of an inner velocity control loop run at 1 KHz by the motor controller EPOS4 (maxon Group, Switzerland) in cascade to an outer PID torque control loop run at the same frequency and implemented in Twincat (BeckhoffAutomation Technologies, Germany). A parallel safety block is implemented to disable the motor in case of dangerous interaction torques resulting from an unexpected event, for example, a shock resulting from a fall of the user. Moreover, safety thresholds are created to prevent the OTS and the flexible shaft from breaking in case of an unexpected peak of torque.

The encoders’ data 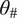 are used to estimate the output torque 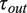 at the level of the physical human–robot interface. The motor position 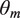 and the hypoid gear position 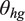 are used to estimate 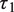 , given the model of the flexible shaft and hypoid gearbox. Then, 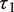 is added to 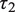 , in output from the PEM block, to obtain 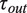 . The latter parameter is controlled by means of a PID to make the output torque converge with the preset value of desired torque 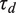 .

The safety and torque control loop are implemented on a computer (CORE i7, 8th generation) with two dedicated cores for real-time communication, running TwinCAT (Beckhoff Automation Technologies) as a communication interface and using EtherCAT (1 kHz sampling frequency) as a communication protocol (K. Langlois et al., 2018). Beckhoff components are used for data acquisition and analog-to-digital conversion.

### 2.3. Experimental evaluation

Experiments were conducted to test the different components of the exoskeleton structure. In an early phase, in vitro tests were performed on the single elements of the RAS for the:

- Flexible shaft characterization;
- OTS static characterization;
- Hypoid gearbox characterization;
- PEM model validation.

**Figure 6.**
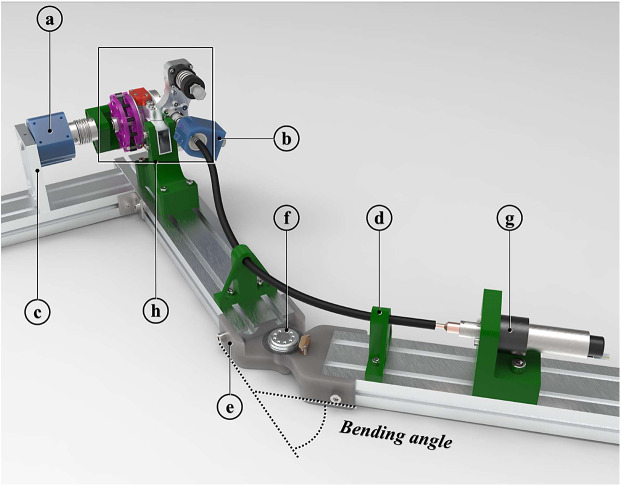
Test bench: (a) 20 Nm torque sensor (ETH Messtechnik, DRBK); (b) 2 Nm torque sensor (ETH Messtechnik, DRBK); (c) grounding plate; (d) 3D printed parts for flexible shaft routing; (e) hinge equipped with encoder; (f) optical encoder (US Digital E6); (g) drive unit; and (h) EEM.

A purposely designed setup was realized (Figure 6). Torque sensors (a and b) were connected to the EEM (h) input and output axes. The latter one was rigidly constrained to the ground through an aluminum plate (c). The drive unit (g) was directly connected to the torque sensor (b) via the Dremel flexible shaft. The 3D printed supports (d) were realized to constrain the flexible shaft path to follow an arc-like trajectory over the hinge (e), whose angular displacement was measured through an additional optical encoder (f).

After the characterization of the different components, a second batch of tests was carried out to evaluate the performance of the whole RAS and to tune the indirect torque controller.

Finally, experiments with participants were performed to evaluate the torque controller in a real-life scenario (De Bock et al., 2020) and to assess the exoskeleton comfort and usability.

#### 2.3.1. Flexible shaft characterization

Flexible shafts are realized by winding multiple layers of harmonic steel wire around a mandrel that constitutes the core of the flexible shaft. The number of layers, the grade and size of the wire, the length of the shaft and, its diameter are all manufacturing parameters that determine the shaft’s mechanical properties. Among those, it is worth noting two interesting ones: (i) a direction-dependent torsional stiffness and (ii) a bending-dependent torsional stiffness. The latter is especially observed for low bending radius: when a torque is applied onto the shaft, the latter tends to deform in a helix-like shape. This effect results in frictional losses and lower efficiency of the torque transfer.

However, since in our design the shaft assumes curved paths and is intended as an element whose elastic properties are exploited for indirect torque control, its torque-deflection/bending characteristic becomes of primary importance (Rodriguez-Cianca et al., 2019).

The test bench in Figure 6 was used to characterize the flexible shaft. The drive unit was controlled in position through the EPOS4, to produce a quasi-static sinusoidal displacement at 0.05 Hz of one end of the shaft. The other end was connected to the EEM via a torque sensor (b). As a result of an angular displacement, a torque on the shaft was measured. To distinguish between the direction-dependant stiffness and the bending-dependent stiffness two testing conditions were designed.

First, the amplitude of the sinusoidal input displacement was varied from 40° to 200° to validate the system characteristics throughout different relevant torque amplitudes. During this test, the shaft was kept straight (bending angle equal to zero). Each sinusoidal displacement was repeated five times.

Then, the stiffness of the flexible shaft was characterized across different bending angles (0°, 20°, and 40°) while keeping the amplitude of the sinusoidal input displacement constant to 200°. Knowing the arc length of the shaft laying between the 3D-printed supports (d), a bending radius 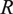 was associated with each bending angle. For each bending radius ( 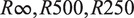 ), the input sinusoidal displacement was repeated five times.

After testing, data points were fitted with a 4th-degree polynomial to derive a model from the torque-deflection data of the shaft.

#### 2.3.2. Output torque sensor static calibration

The test setup in Figure 6 was also used to calibrate the OTS described in Section 2.2.2. The calibration reference instrument was a commercial torque sensor a) (20 Nm torque sensor, ETH Messtechnik, DRBK torque transducers, Figure 6), connected on one side, to the output axis of the RAS and grounded on the other side. Twelve loading/unloading torque cycles were applied on the OTS. Static torques ranging from −10 to 10 Nm were produced to stress the OTS-sensitive element, that is the carbon fiber beam. The RAS magnetic encoders (Figure 4a–c) were exploited to measure the beam deflection.

After testing, data were fitted with a linear function to derive the OTS calibration curve.

#### 2.3.3. Hypoid gearbox characterization

Hypoid transmission efficiency is a parameter that has shown high sensitivity to a multitude of factors such as the manufacturing accuracy of the gear and pinion, the assembly tolerances, and the operation conditions (Kolivand et al., 2010). An a priori estimation of the input–output torque relationship of the hypoid transmission is challenging without the knowledge of the aforementioned factors. Therefore, a characterization is deemed necessary.

Thanks to the test setup in Figure 6, it was possible to measure the torque 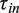 in input to the hypoid pinion and, at the same time, the torque 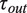 in output from the hypoid gear. The transmission efficiency was calculated as 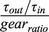 .

The setup shown in Figure 6 did not allow to measure back-driving torques, since the output of the gear was rigidly connected to ground. The torque necessary to back-drive the whole RAS will be computed in the context of the in vivo experiments.

The PEM mechanism was disconnected from the EEM so to avoid potential interfering input to the measurement system. A quasi-static sinusoidal torque at 0.05 Hz with amplitude ranging from 0.1 to 0.6 Nm, was generated with the drive unit and transferred to the input axis (pinion) of the hypoid transmission, where it was measured by the torque sensor (b). Concurrently, sensor (a) gauged the output torque. For each torque amplitude, two sinusoidal waves were generated.

#### 2.3.4. PEM model validation

**Figure 7.**
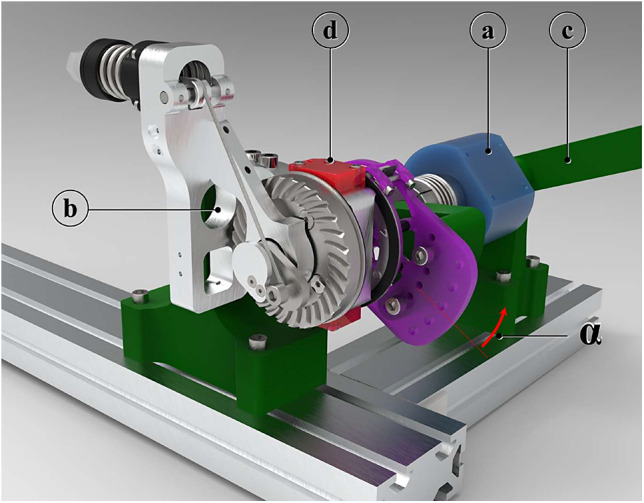
Test bench setup realized to validate the PEM setup. (a) 20 Nm torque sensor (ETH Messtech- nik, DRBK torque transducers); (b) housing of the hypoid pinion; (c) lever arm; and (d) magnetic encoder.

The test setup shown in Figure 6, was adapted to make the rotation of the EEM output axis possible. For this reason, the grounding plate was substituted with a lever arm (Figure 7). To validate the theoretical model for the PEM, the lever-arm (c) was manually rotated in a quasi-static fashion, making the angle 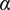 vary slowly. In the meantime, the torque necessary to rotate the exoskeleton link was recorded with the torque sensor (a). The encoder (d) embedded in the EEM was used to gauge the angle 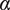 . It is worth noting that the pinion of the hypoid transmission was dismounted from its housing (b), so to remove one interfering source of friction affecting the torque generated by the PEM and thus the validation. Five loading and unloading cycles were done.

#### 2.3.5. In vitro assessment of the RAS performance

The test bench setup shown in Figure 6 was used for all the experiments conducted to tune the control architecture introduced in Section 2.2.3 and to assess the performance of the RAS. The flexible shaft torque-deflection characteristic and the hypoid gearbox input–output relationship were used to estimate the RAS output torque. Simultaneously the output torque was measured with the commercial torque sensor (a) (Figure 6), mounted between the RAS output axis and ground.

A first experiment was conducted to tune the PID parameters of the main torque loop of the controller. The Ziegler–Nichols approach was used to minimize the rise time of the system in following a desired step in torque of 5 Nm amplitude.

Then, the RAS bandwidth was studied using the approach presented in (Rodriguez-Cianca et al., 2019). A multi-sine signal with a flat spectrum from 0.1 to 10 Hz and variable amplitude (0–5 Nm) was selected as a desired output torque 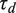 for the RAS. The actual output torque 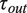 and the desired set-point torque 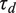 , were used to estimate the transfer function of the RAS, 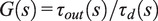 . The system identification toolbox implemented in MATLAB (MathWorks, USA) was used to obtain 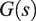 .

### 2.4. In vivo assessment of the AE4W performance

Seven healthy male participants (age: 29 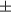 3 years, height: 1.80 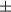 0.03 m, weight: 78.0 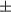 5 kg) were included in an experiment to assess the real-life performance of the AE4W. The aim of the experiment was twofold: (i) verify the exoskeleton’s kinematic compatibility and comfort; (ii) verify the suitability of the RAS for assisting real-life human tasks. Before the start of the experiment, all participants signed the informed consent. The experimental protocol received approval from the local ethical commission (Vrije Universiteit Brussel and Universitair Ziekenhuis Brussel, BUN: 143201941463).

All participants performed three tasks with (Exo) and without exoskeleton (NoExo). After completion of each task, the rating of perceived exertion (RPE) and the comfort at the level of the shoulder were collected. To determine the RPE, Borg’s 1–10 RPE scale was used (Borg, 1982).

#### 2.4.1. Test 1: Active ROM test

**Figure 8.**
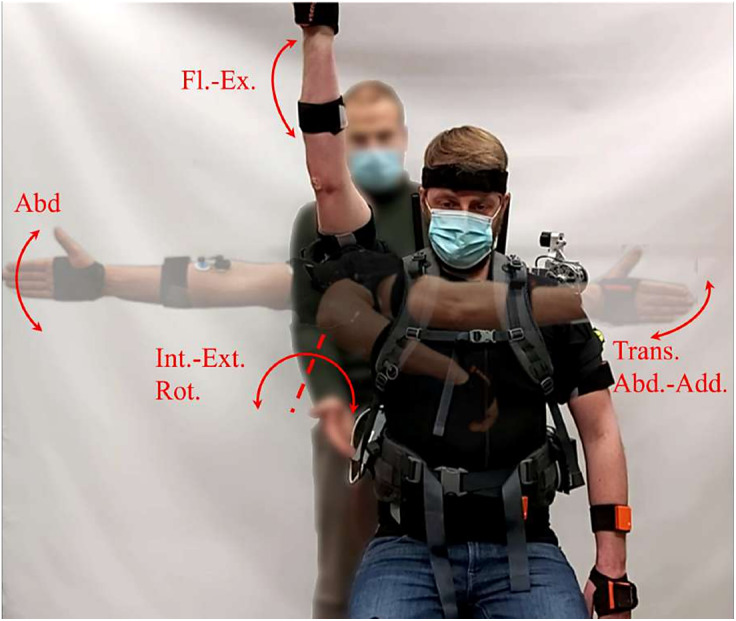
Active ROM test. The arrows highlight the different movements performed during the test.

The ROM test was executed in four isolated directions; Flexion-extension (Fl-Ex), Frontal abduction (Abd), Transverse ab-adduction (Trans. Abd – Trans. Add), Internal–external rotation (Int. Rot – Ext. Rot). All participants autonomously moved the dominant arm three times in each direction until the maximal shoulder ROM was reached (Dutton, 2014) (Figure 8). To prevent compensatory movements with the rest of the body, an expert movement scientist supervised the execution of the tests.

This test was executed with and without AE4W so to assess the compatibility of the exoskeleton’s kinematic structure. Since exoskeleton assistance can affect the shoulder ROM (van der Have et al., 2021), the AE4W was powered off during the active ROM tests. Moreover, the PEM was disconnected from the RAS to avoid interference because of the passively generated assistance.

The MTw Awinda (Xsens, the Netherlands) was used for user motion tracking.

#### 2.4.2. Test 2: Constant torque support during lifting

**Figure 9.**
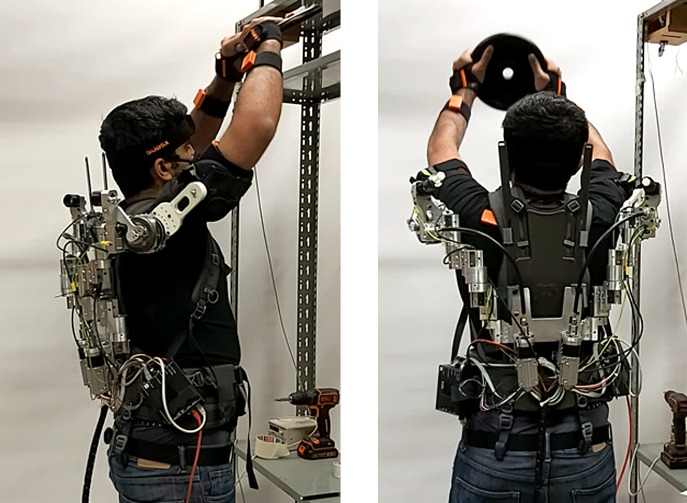
Left: constrained lifting task. Right: free lifting task.

This task was designed to test the ability of the torque controller to deliver a constant supportive torque of 5 Nm during a dynamic lifting movement. The participants lifted a 5-kg weight from hip to overhead height for five times (Figure 9). Each motion started with the subjects standing in front of a rack while holding the weight in front of the pelvis. The motion ended when the weight was placed on a shelf, positioned 50 cm above the level of the acromion (van der Have et al., 2021). The actuator was disabled when returning to the starting position. During this shoulder extension movement, participants backdrove the RAS. The sensors of the AE4W were used for data acquisition.

#### 2.4.3. Test 3: Ballistic active support during cyclic lifting

The participants lifted and lowered a 5-kg weight five times at a self-selected speed without interruption between the repetitions. They were instructed to keep their elbow joint fixed in a comfortably extended position. The task started and ended with the weight in front of the pelvis.

AE4W was controlled to deliver a ramp in torque to assist the elevation of the users’ arms in a ballistic manner. The RAS functioning was enabled at 30° of elevation angle of the user’s shoulder joint. Together with the PEM, AE4W delivered a peak of 12 Nm at 80°. When lowering the weight, the RAS was disabled and the shoulder extension movement was only passively supported by the PEM. This task was executed to test the AE4W controller in real-life cyclic tasks, therefore the shape of the assistive torque profile was arbitrarily chosen.

The sensors of the AE4W were used to track the exoskeleton’s behavior and to estimate the users’ shoulder elevation angle.

Thirty-five lifts were analyzed. Each lift was resampled to 100 data points. The measured peak of assistance was used to align each different lift in time. The time instant when the participants received the maximum of the assistance was set as zero (Figure 16).

### 2.5. Statistical analysis

Descriptive statistics were used to represent the data collected through test bench experiments. Means accompanied with confidence intervals of 95% were reported for the measured data (Accoto et al., 2018). When models were employed, the fitting accuracy was assessed by the Root Mean Square Error (RMSE) and the normalized RMSE (NRMSE). The latter was defined as:
(2.5)

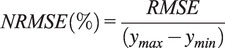

with 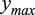 and 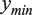 being, respectively, the maximum and the minimum value of the observation.

When a comparison across experimental conditions was required, normality of the distribution was checked using the Shapiro–Wilk test, and a one-way ANOVA analysis was performed. Paired sample *T*-tests were used for pairwise comparisons. When normality was violated, the Friedman and Wilcoxon signed-rank tests were used to compare data across conditions. The significance level for all statistical tests was set at 0.05.

## 3. Results

### 3.1. In vitro tests

#### 3.1.1. Flexible shaft torque-deflection characteristics

**Figure 10.**
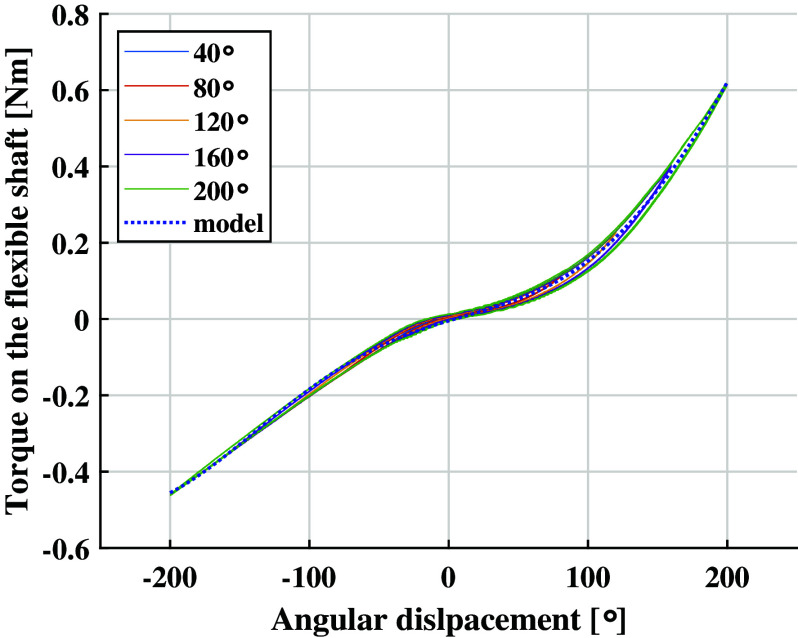
Deflection-dependent characteristics of the flexible shaft. Not appreciable differences are noted for different torque amplitudes.

The flexible shaft exhibited a direction-dependent stiffness (Figure 10). Positive angular displacements brought higher torques. Considering the profile obtained with a deflection of 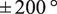 , the torque reached for positive displacements was ~27% higher than the torque reached for negative displacements.

The experimental data could be fit by a fourth-degree polynomial 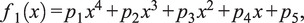 whose coefficients 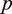 were 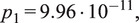 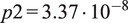 , 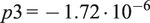 , 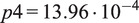 , 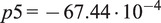 and 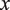 represented the angular displacement. The model predictions showed an RMSE of 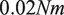 , leading to an NRMSE of 1.4%. This corresponded to a mean accuracy of 98.6% in estimating the torque on the flexible shaft.

**Figure 11.**
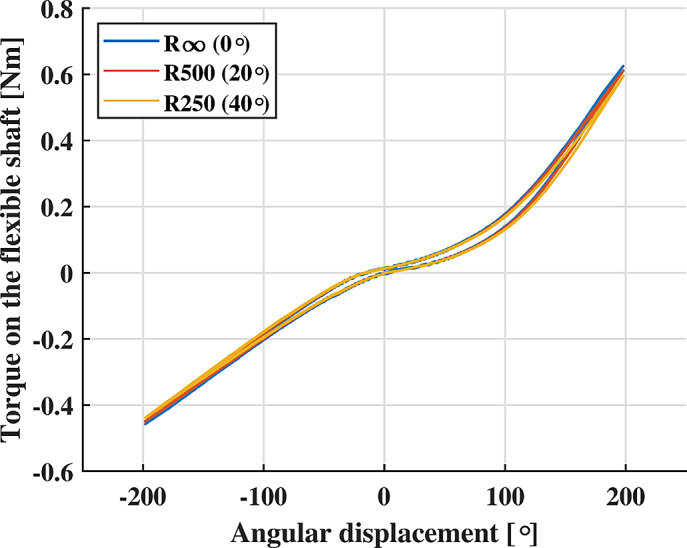
Bending-dependent characteristics of the flexible shaft. Not appreciable differences are found between bending conditions 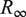 and 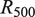 and, between 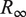 and 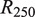 .

The flexible shaft exhibits a moderate bending-dependent stiffness (Figure 11). The ANOVA analysis showed differences between the bending conditions 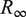 , 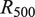 and, 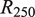 ( 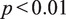 ). The *T*-test confirmed statistical difference between the bending conditions 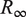 and 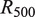 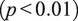 and, between 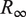 and 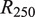 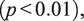 being the latter the most appreciable difference. It was also possible to validate the model 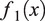 , obtained for the flexible shaft configuration 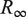 , with the experimental data found for the configuration 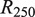 . Analogously to the straight configuration of the flexible shaft 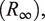 the model predictions for the bent configurations showed an RMSE of 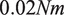 . It is worth noting that this torque deviation was about the same order of magnitude as the RMSE related to the straight model. Therefore, a model correction compensating for the shaft bending radius was not necessary.

#### 3.1.2. OTS calibration curve

The OTS calibration curve confirmed the linear relationship between torque and carbon fibre beam deflection (Ducastel et al., 2021). The experimental data were fit by a first degree polynomial 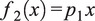 , whose coefficient 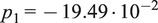 and 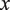 represented the torque input. The model predictions showed an RMSE of 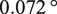 , leading to an NRMSE of 2%. This corresponded to a mean accuracy of 98%. By inverting 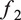 , the sensitivity of the OTS was computed as 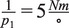 . A torque increment of 0.25 Nm would be measured as a consequence of a beam deflection 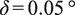 , the latter corresponding to the resolution of the magnetic encoders embedded in the RAS (Figure 4, a–c).

#### 3.1.3. Hypoid gearbox input–output torque relationship

The hypoid transmission input and output torques were linearly correlated. The torque ratio 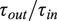 was thus constant and equal to 10.7. This corresponded to an efficiency of 71%.

#### 3.1.4. PEM model validation

**Figure 12.**
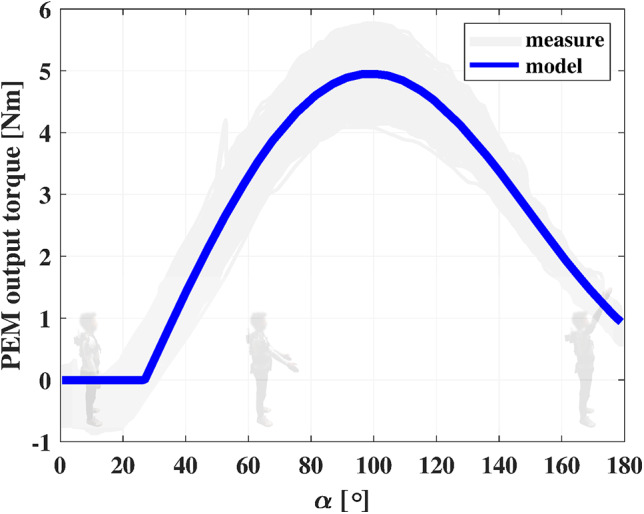
PEM torque-angle characteristic. For low shoulder elevation angles 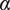 , the delivered assistance is ~0 Nm. The peak of assistance occurs for 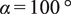 .

The output torque profile generated with the PEM is shown in Figure 12. The measured hysteresis cycle testified the presence of Coulomb friction in the mechanism. Because of that, part of the energy stored in the spring during an arm extension was not returned to the user amid an arm flexion. The lost energy was computed by calculating the area enclosed by the hysteresis cycle. Out of the 9.5 J needed to load the spring, 2.0 J were dissipated after a cycle. Despite the PEM having a reduced number of sliding/rolling components in comparison with its previous implementation introduced in (van der Have et al., 2021), nearly 20% of the energy was dissipated.

The accuracy of the model was evaluated through its normalized root mean square deviation from the experimental data. An NRMSE of 18% was calculated, yielding an overall estimated accuracy of 82%.

#### 3.1.5. RAS step response and bandwidth estimation

**Figure 13.**
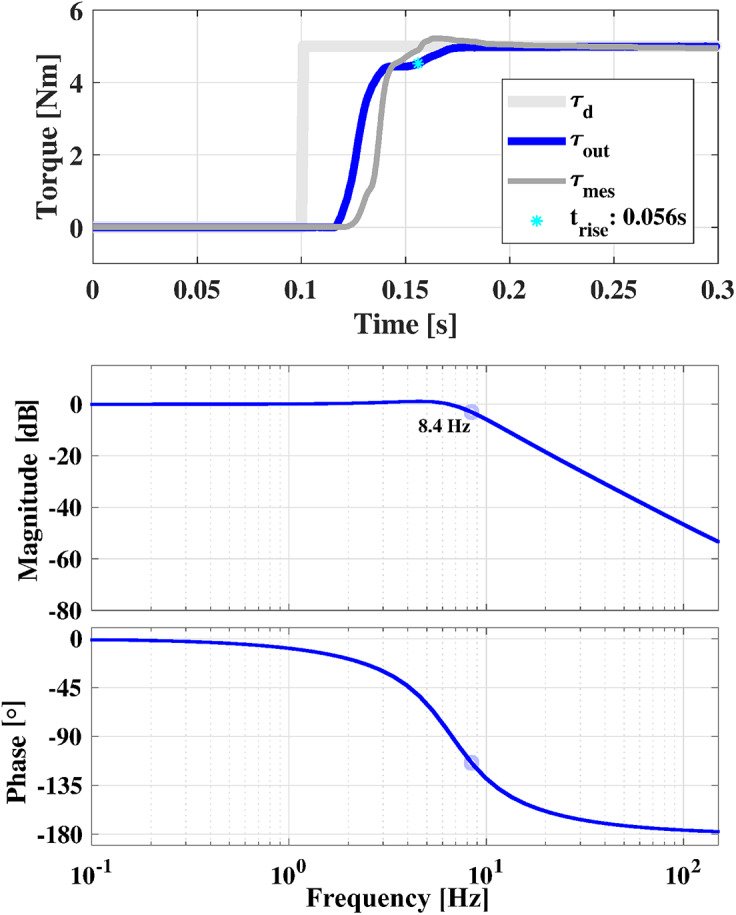
Top: step response of the RAS. The estimated output torque 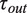 is compared with the measured output torque 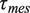 and the desired torque 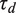 . The rise time of the RAS is highlighted by the light blue star. Bottom: Bode plot of the transfer function 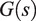 , obtained starting from the multisine desired torque signal and the actual output of the RAS.

The step response of the RAS is shown in Figure 13. Overall, the controlled torque 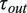 reached 95% of the desired output torque 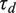 in 56 ms. The rise time, defined as the time needed for the RAS response to rise from 5% to 95% of its final value, amounted to 37 milliseconds.

The output torque 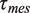 measured by the torque sensor (a, Figure 6), slightly deviated from the estimated output torque 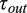 . This was because of two factors. First, the calibration curve of the OTS did not fit the measured data with an accuracy of 100%. Secondly, the sensitivity of the OTS was lower than the sensitivity of the commercial torque sensor used in this experiment.

A small rise time of the RAS step response may indicate a large bandwidth of the system. The latter was estimated through the experiment described in Section 2.3. The Bode plot of transfer function 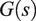 of the RAS is reported in Figure 13. The 3 dB bandwidth of the system corresponded to 8.4 Hz.

### 3.2. In vivo tests

#### 3.2.1. Test 1: active ROM test

**Figure 14.**
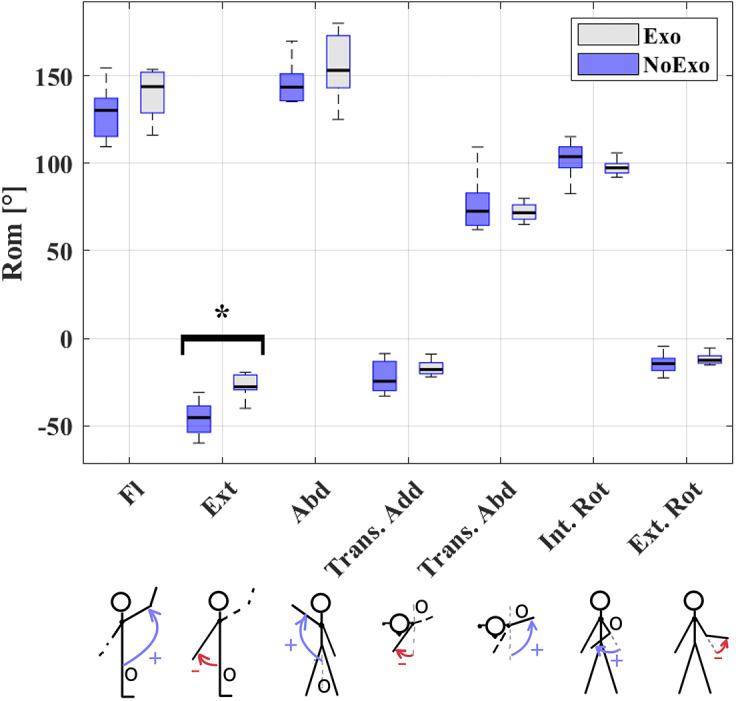
ROM data for flexion-extension, abduction. Transverse ab-/adduction. The following convention was used: flexion, frontal abduction, transverse abduction, and internal rotation were considered positive movements; extension, transverse adduction, and external rotation were considered negative movements. The horizontal lines in the middle of each box represent the median, while the whiskers report the minimum and the maximum measured joint angles. Top and bottom lines of each box represent the 75th and 25th percentile, respectively. The star symbol indicates statistically significant differences.

The shoulder ROM in each of the measured directions is represented in (Fig 14).

The AE4W did not significantly reduce anatomical ROM in flexion, abduction, transverse ab-/adduction and internal–external rotation. However, the shoulder extension ROM was significantly reduced from −44° (std: ±12°) without exoskeleton to −28° (std: ±6°) ( 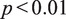 ) with AE4W.

The exoskeleton also affected the RPE and comfort scores. Wearing the exoskeleton significantly increased the RPE score. Without exoskeleton, participants rated the ROM test as “Easy” (3.0 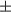 1.6), while the score with AE4W was “Moderate” (4.0 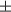 1.1) ( 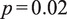 ). Also, when performing the active ROM test with the exoskeleton, participants experienced a lower level of comfort at the shoulder (71.3 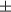 14.6 mm) compared with the condition without exoskeleton (87.3 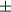 9.5 mm) ( 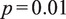 ).

#### 3.2.2. Test 2: Constant torque support during lifting

**Figure 15.**
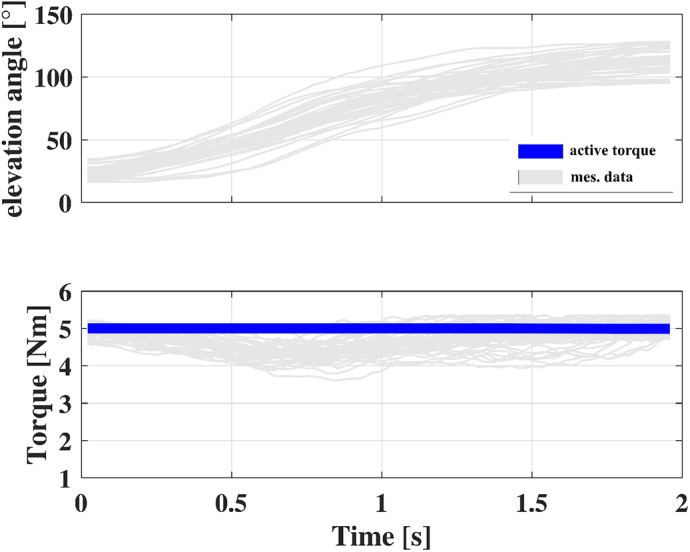
Top: shoulder joint trajectory measured with the AE4W; Bottom: amount of assistance delivered to the user during the elevation phase of the arms. Light grey lines represent each acquired trajectory. The blue line represents the desired torque.

Thirty-five lifts were analyzed and each lift was resampled to 100 data points. The mean duration of a lifting movement and the AE4W assistance were determined (Figure 15).

During this test, the AE4W controller managed to keep a quasi-constant support throughout the entire lifting movement. On average, the RMSE between the set-point torque 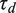 and the delivered torque 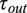 was 0.4 Nm. The NRMSE was of 7.5%. The minimum average torque was delivered at approximately 30% of the lift, where the shoulder joint acceleration peaked. In this instant, the minimum average torque delivered was of 4.3 Nm. The maximum average torque delivered during the lift was 5.1 Nm. The participants moved with an average velocity of 87 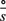 , peaking at about 50% of the motion and reaching up to 145 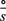 .

The mean backdriving torque of the RAS was measured while participants were lowering their arms to come back to the task’s starting position. An average value of 0.6 Nm (±1 Nm) was gauged. The average velocity of the arm extension movement was −81 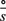 (max: −143 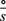 ).

The AE4W did not significantly affect the comfort at the level of the shoulder. However, it did affect the RPE. Without exoskeleton an RPE-score of 4.6 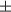 1.6 (“Challenging”) was observed. Wearing the exoskeleton significantly reduced this score to 3.4 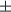 0.9 (“Moderate”) (p = 0.04).

#### 3.2.3. Test 3: Ballistic active support during cyclic lifting

**Figure 16.**
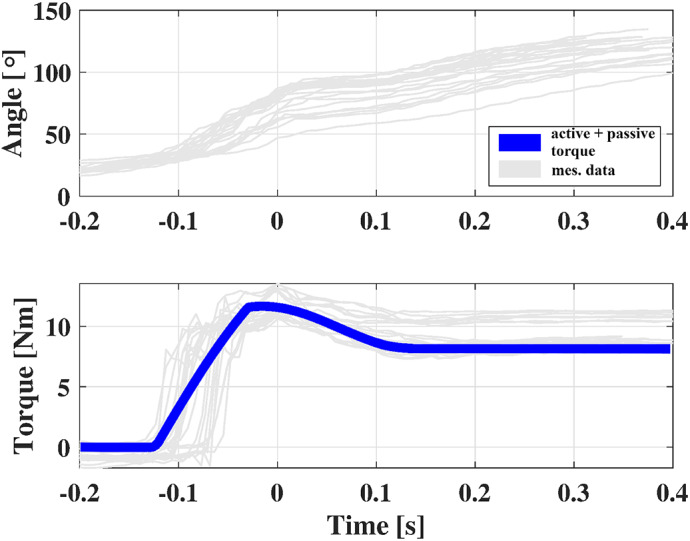
Up: shoulder joint trajectory measured with the AE4W; Down: amount of assistance delivered to the user during the elevation phase of the arms. Light gray lines represent each measured data. The blue line represents the set-point desired torque.

AE4W managed to provide up to 12 Nm during the execution of the cyclic task (Figure 16). On average, the RMSE between the set-point torque 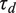 and the delivered torque 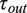 was 1.9 Nm. The NRMSE was of 15.0%. The participants moved with an average velocity of 153 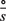 , reaching up to 184 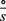 maximally.

The AE4W significantly improved the perceived comfort at the level of the shoulder. Without exoskeleton, subjects perceived less comfort (58 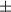 20 mm) than with AE4W (71 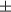 9 mm) 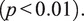 This feeling was reflected in a significant reduction of the RPE from “Challenging” (4.6 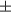 2.3) without exoskeleton, to “Moderate” with exoskeleton (3.6 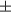 1.7) 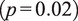 .

## 4. Discussion

The objective of this work was to develop an active shoulder exoskeleton meant to assist the workers during the execution of occupational tasks. First of all, the problem of achieving kinematic compatibility with the motion of the human shoulder joint was considered. A structural study (Rossini et al., 2021c) was conducted to enumerate the possible exoskeleton kinematic chains not preventing the biological shoulder motion. Six kinematically equivalent chains were found but only one was chosen for further implementation considering ergonomics and practical considerations, such as the possibility to reduce the frontal footprint of the exoskeleton. The compatibility of AE4W kinematic structure was also assessed through an experiment with young healthy participants. An active ROM test with and without exoskeleton showed that the AE4W structure did not reduce the natural ROM of the shoulder in any of the direction of motion, but shoulder extension. Shoulder over-extension is not required during occupational tasks (Huysamen et al., 2020). Therefore, the majority of the workers would not experience ROM limitations when wearing the AE4W. Finally, the AE4W increased perceived exertion of the active ROM test from “Easy” to “Moderate.” These data were congruent with the reported reduced comfort at the level of the shoulder. It is worth noting that the strapping pressure of the physical human–robot interfaces was set according to the participants’ preferences during the initial donning phase of the AE4W. The reported hindrance at the level of the arm cuff indicates that adjusting the strapping pressure during the experiment, as proposed by (K. Langlois et al., 2020), may result in a more comfortable wearing experience.

Specific experiments were conducted to characterize the RAS components. The flexible shaft torque-angle characteristics were found and modeled so that, once the angular deflection of the shaft was measured, the estimation of the transferred torque was possible. The shaft exhibited a mild bending-dependent stiffness change. However, different from the variations of the flexible shaft properties measured in Rodriguez-Cianca et al. (2019)), such a change was not sufficiently marked to require further modeling of this phenomenon. Consequently, a simplified model of the shaft was employed for different bending configurations, leading to an average torque estimation accuracy of 98.6%.

To estimate the output torque delivered by the RAS, the hypoid gearbox was also characterized. The modeling of its behavior was confined to the specific application scenarios tested in this work, that is, assisting the shoulder flexion movements by providing elevation torques. A linear relationship between the input and the output torque of the hypoid gearbox was found and an efficiency of about 70% was measured. Despite the low value, the typical efficiency ranges of hypoid gearboxes are 50%–90% (Radzevich, 2016).

The need for the implementation of a custom-made torque sensor, the OTS, in the RAS arose from the analysis of the off-the-shelf commercial torque sensors. The latter ones normally have high stiffness and high resolution, unnecessary characteristics for the monitoring of the human–robot interaction forces/torques. A compact design for the OTS was presented. Its functioning was based on the measurement of the deflection of an elastic element, that is a carbon fiber beam. The calibration of the sensor showed a sensitivity of 5 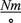 . Its theoretical resolution was 0.25 Nm, as derived from the resolution of the encoders. The sensor was then employed in the RAS safety control loop, as a redundant source of human–robot interaction torque monitoring.

The PEM has two purposes: (i) boost the RAS performance in terms of the amount of delivered torque to the user; (ii) assure a passive functioning of the AE4W. The latter aspect is important for safety reasons, preventing an abrupt fall in torque in case of a power outage or interruption of the motor operation.

In vitro tests showed that the RAS was fast enough to provide an effective human–robot interaction. A rise time of 37 ms in providing a step in torque of 5 Nm amplitude and an estimated RAS bandwidth of 8 Hz is recommended requirement for actuators used in human–robot interaction applications (Kong et al., 2009).

The suitability of the RAS for assisting occupational tasks was corroborated by in vivo experiments. In both Test 2 and 3, participants executed the task at the lifting speed of their preference. However, the positioning nature of the first task automatically leads to a lower shoulder elevation speed (87 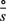 ) that almost doubled during the execution of the second task (153 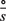 ). Despite the peak velocities reached during Tests 2 and 3 were higher than what was reported in the requirement list of (Huysamen et al., 2020), the AE4W successfully managed to provide assistance. The constant support control strategy showed an accuracy of 92.5% in tracking the desired output torque while the ballistic support showed an accuracy of 85%.

During Test 2, the backdriving torque of the RAS was also measured with the OTS. A peak value of 0.7 Nm was found for extension velocities up to −143 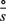 . Such a value can be considered low in comparison to other work reporting backdriving torques up to 10 Nm for actuators used in human–robot applications (Accoto et al., 2014; Ducastel et al., 2021).

Finally, the use of AE4W led to a reduced RPE in both lifting tasks. The AE4W reduced the perceived exertion during the task from “Challenging” without exoskeleton, to “Moderate.”

## 5. Study limitation and future work

The study aimed at developing an active occupational shoulder exoskeleton and validating its mechanical performance both with in vitro and in vivo tests. Mechanical parameters related to the device functioning but also subjective parameters, such as RPE and comfort at the level of the shoulder, were collected. The results of the pilot tests showed the potential of the AE4W. However, the device’s weight could negatively affect metabolic cost and mechanical joint work, for example, hip or knee (De Bock et al., 2022; Kim et al., 2018). Indeed, the current prototype of AE4W is relatively heavy when compared with existing active shoulder exoskeletons, such as the Shoulder-SideWINDER, weighing 6.4 kg (Park et al., 2021) and the S700, weighing 7.4 kg (Sänger et al., 2022). Therefore, future efforts will be directed toward the optimization of the weight of the device, including the power electronics. Moreover, a more sophisticated control architecture is deemed necessary to allow motion intention estimation and optimization of the assistive strategies. Only then, an extensive evaluation of the device will be carried out to investigate its task-dependent positive or negative effect on the user’s musculoskeletal loading.

## 6. Conclusion

AE4W is one of the first active occupational shoulder exoskeletons to be conceived as a portable device worn by the user. A design methodology validated with in vitro and in vivo experiments has been proposed for the exoskeleton kinematics, actuation system, and control architecture. Results have shown the performance and versatility of AE4W.

## Data Availability

Data can be made available to interested researchers upon request by email to the corresponding author.

## References

[r1] Accoto D, Rossini M, Valentini S and Portaccio I (2018) A novel sensor for measuring the inner pressure of catheters for clinical use. IEEE Sensors Journal 18(9), 3564–3571.

[r2] Accoto D, Sergi F, Tagliamonte NL, Carpino G, Sudano A and Guglielmelli E (2014) Robomorphism: A nonanthropomorphic wearable robot. IEEE Robotics & Automation Magazine 21(4), 45–55.

[r3] Babič J, Laffranchi M, Tessari F, Verstraten T, Novak D, Šarabon N, Ugurlu B, Peternel L, Torricelli D and Veneman JF (2021). Challenges and solutions for application and wider adoption of wearable robots. Wearable Technologies 2, e14. 38486636 10.1017/wtc.2021.13 PMC10936284

[r4] Bevan S (2015) Economic impact of musculoskeletal disorders (MSDs) on work in Europe. Best Practice & Research Clinical Rheumatology 29(3), 356–373. 10.1016/j.berh.2015.08.002 26612235

[r5] Blanco A, Catalán JM, Martínez-Pascual D, García-Pérez JV and García-Aracil N (2022) The Effect of an Active Upper-Limb Exoskeleton on Metabolic Parameters and Muscle Activity During a Repetitive Industrial Task. IEEE Access.

[r6] Borg GA (1982) Psychophysical bases of perceived exertion. Medicine and Science in Sports and Exercise 14(5), 377–381. 7154893

[r7] Crea S, Beckerle P, De Looze M, De Pauw K, Grazi L, Kermavnar T, Masood J, O’Sullivan LW, Pacifico I, Rodriguez-Guerrero C, Vitiello N, Ristić-Durrant D and Veneman J (2021). Occupational exoskeletons: A roadmap toward large-scale adoption. Methodology and challenges of bringing exoskeletons to workplaces. Wearable Technologies 2, e11. 10.1017/wtc.2021.11 38486625 PMC10936259

[r8] De Bock S, Ampe T, Rossini M, Tassignon B, Lefeber D, Rodriguez-Guerrero C, Roelands B, Geeroms J, Meeusen R and De Pauw K (2023). Passive shoulder exoskeleton support partially mitigates fatigue-induced effects in overhead work. Applied Ergonomics 106, 103903. 36148702 10.1016/j.apergo.2022.103903

[r9] De Bock S, Ghillebert J, Govaerts R, Elprama SA, Marusic U, Serrien B, Jacobs A, Geeroms J, Meeusen R and De Pauw K (2020) Passive shoulder exoskeletons: More effective in the lab than in the field? IEEE Transactions on Neural Systems and Rehabilitation Engineering 29, 173–183. 10.1109/TNSRE.2020.3041906 33264094

[r10] De Bock S, Ghillebert J, Govaerts R, Tassignon B, Rodriguez-Guerrero C, Crea S, Veneman J, Geeroms J, Meeusen R and De Pauw K (2022a) Benchmarking occupational exoskeletons: An evidence mapping systematic review. Applied Ergonomics 98, 103582. 34600307 10.1016/j.apergo.2021.103582

[r11] De Bock S, Rossini M, Lefeber D, Rodriguez-Guerrero C, Geeroms J, Meeusen R and De Pauw K (2022) An occupational shoulder exoskeleton reduces muscle activity and fatigue during overhead work. IEEE Transactions on Biomedical Engineering. 69(10), 3008–3020. 35290183 10.1109/TBME.2022.3159094

[r12] De Looze MP, Bosch T, Krause F, Stadler KS and O’Sullivan LW (2016) Exoskeletons for industrial application and their potential effects on physical work load. Ergonomics 59(5), 671–681. 26444053 10.1080/00140139.2015.1081988

[r13] De Vries A and De Looze M (2019) The effect of arm support exoskeletons in realistic work activities: A review study. Journal of Ergonomics 9(4), 1–9.

[r14] de Vries A, Murphy M, Könemann R, Kingma I and de Looze M (2019) The amount of support provided by a passive arm support exoskeleton in a range of elevated arm postures. IISE Transactions on Occupational Ergonomics and Human Factors 7(3–4), 311–321.

[r15] Ducastel V, Langlois K, Rossini M, Grosu V, Vanderborght B, Lefeber D, Verstraten T and Geeroms J (2021) Smarcos: Off-the-shelf smart compliant actuators for human-robot applications. Actuators 10(11), 289.

[r16] Dutton M (2014) Range of motion. Introduction to Physical Therapy and Patient Skills. New York, NY: McGraw-Hill Education.

[r17] Ebrahimi A (2017) Stuttgart exo-jacket: An exoskeleton for industrial upper body applications. 2017 10th International Conference on Human System Interactions (HSI), 258–263. IEEE.

[r18] EU-OSHA (2020) *Work-Related Musculoskeletal Disorders: Prevalence, Costs and Demographics in the eu.* Publications Office. 10.2802/66947

[r19] Fraco, Mawashi, Quebec, Canada (2020) https://www.fraco.com/en/documents/Fraco_Exoskeleton.pdf

[r20] Gogu G (2002) Families of 6r orthogonal robotic manipulators with only isolated and pseudo-isolated singularities. Mechanism and Machine Theory 37(11), 1347–1375.

[r21] Gopura R, Bandara D, Kiguchi K and Mann GK (2016) Developments in hardware systems of active upper-limb exoskeleton robots: A review. Robotics and Autonomous Systems 75, 203–220.

[r22] Gopura RARC, Kiguchi K and Bandara DSV (2011) A brief review on upper extremity robotic exoskeleton systems. In 2011 6th international Conference on Industrial and Information Systems, 346–351. IEEE.

[r23] Govaerts R, Tassignon B, Ghillebert J, Serrien B, De Bock S, Ampe T., El Makrini I, Vanderborght B, Meeusen R and De Pauw K (2021) Prevalence and incidence of work-related musculoskeletal disorders in secondary industries of 21st century europe: A systematic review and meta-analysis. BMC Musculoskeletal Disorders 22(1), 1–30. 34465326 10.1186/s12891-021-04615-9 PMC8408961

[r24] Grazi L, Trigili E, Caloi N, Ramella G, Giovacchini F, Vitiello N and Crea S (2022) Kinematics-based adaptive assistance of a semi-passive upper-limb exoskeleton for workers in static and dynamic tasks. IEEE Robotics and Automation Letters 7(4), 8675–8682.

[r25] Grazi L, Trigili E, Proface G, Giovacchini F, Crea S and Vitiello N (2020) Design and experimental evaluation of a semipassive upper-limb exoskeleton for workers with motorized tuning of assistance. IEEE Transactions on Neural Systems and Rehabilitation Engineering 28(10), 2276–2285. 32755865 10.1109/TNSRE.2020.3014408

[r26] Howard J, Murashov VV, Lowe BD and Lu M-L (2020) Industrial exoskeletons: Need for intervention effectiveness research. American Journal of Industrial Medicine 63(3), 201–208. 31828844 10.1002/ajim.23080

[r27] Huysamen K, Power V and O’Sullivan L (2020) Kinematic and kinetic functional requirements for industrial exoskeletons for lifting tasks and overhead lifting. Ergonomics,63(7), 818–830. 32320343 10.1080/00140139.2020.1759698

[r28] Hyun DJ, Bae K, Kim K, Nam S and Lee D-h (2019) A light-weight passive upper arm assistive exoskeleton based on multi-linkage spring-energy dissipation mechanism for overhead tasks. Robotics and Autonomous Systems 122, 103309.

[r29] Kim S, Nussbaum MA, Mokhlespour Esfahani MI, Alemi MM, Alabdulkarim S and Rashedi E (2018) Assessing the influence of a passive, upper extremity exoskeletal vest for tasks requiring arm elevation: Part I – “Expected” effects on discomfort, shoulder muscle activity, and work task performance. Applied Ergonomics 70, 315–322. 10.1016/j.apergo.2018.02.025 29525268

[r30] Kolivand M, Li S and Kahraman A (2010). Prediction of mechanical gear mesh efficiency of hypoid gear pairs. Mechanism and Machine Theory 45(11), 1568–1582.

[r31] Kong K, Bae J and Tomizuka M (2009) Control of rotary series elastic actuator for ideal force-mode actuation in human–robot interaction applications. IEEE/ASME Transactions on Mechatronics 14(1), 105–118.

[r32] Langlois K, Rodriguez-Cianca D, Serrien B, De Winter J, Verstraten T, Rodriguez-Guerrero C, Vanderborght B and Lefeber D (2020) Investigating the effects of strapping pressure on human-robot interface dynamics using a soft robotic cuff. IEEE Transactions on Medical Robotics and Bionics 3(1), 146–155.

[r33] Langlois K, van der Hoeven T, Cianca DR., Verstraten T, Bacek T, Convens B, Rodriguez-Guerrero C, Grosu V, Lefeber D and Vanderborght B (2018) Ethercat tutorial: An introduction for real-time hardware communication on windows [tutorial]. IEEE Robotics & Automation Magazine 25(1), 22–122.

[r34] Langlois KP (2022) *Investigation of physical human-robot interfaces for exoskeletons* [Doctoral dissertation, Vrije Universiteit Brussel].

[r35] Lorenz WA, Peshkin MA and Colgate JE (1999) New sensors for new applications: Force sensors for human/robot interaction. In Proceedings 1999 IEEE International Conference on Robotics and Automation (Cat. No. 99CH36288C), 4, 2855–2860.

[r36] Maurice P, Čamernik J, Gorjan D, Schirrmeister B, Bornmann J, Tagliapietra L, Latella C, Pucci D, Fritzsche L, Ivaldi S, et al (2019) Evaluation of paexo, a novel passive exoskeleton for overhead work. Computer Methods in Biomechanics and Biomedical Engineering 22(sup1), S448–S450.

[r37] Menegon FA and Fischer FM (2012) Musculoskeletal reported symptoms among aircraft assembly workers: A multifactorial approach. Work 41(Supplement 1), 3738–3745. 22317290 10.3233/WOR-2012-0088-3738

[r38] Moeller T, Krell-Roesch J, Woll A and Stein T (2022) Effects of upper-limb exoskeletons designed for use in the working environment—a literature review. Frontiers in Robotics and AI 9, 858893. 35572378 10.3389/frobt.2022.858893 PMC9099018

[r39] Mullan J, Vargas Llave O and Wilkens M (2017) *Working Conditions of Workers of Different Ages*: European Working Conditions Survey 2015.

[r40] Näf MB, Junius K, Rossini M, Rodriguez-Guerrero C., Vanderborght B and Lefeber D (2018) Misalignment compensation for full human-exoskeleton kinematic compatibility: State of the art and evaluation. Applied Mechanics Reviews 70(5), 050802.

[r41] Nasr A, Bell S and McPhee J (2023a) Optimal design of active-passive shoulder exoskeletons: A computational modeling of human-robot interaction. Multibody System Dynamics 57(1), 73–106.

[r42] Nasr A, Dickerson CR and McPhee J (2023b) Experimental study of fully passive, fully active, and active–passive upper-limb exoskeleton efficiency: An assessment of lifting tasks. Sensors 24(1), 63. 38202925 10.3390/s24010063 PMC10780908

[r43] Nasr A, Hunter J, Dickerson CR and McPhee J (2023c) Evaluation of a machine-learning-driven active–passive upper-limb exoskeleton robot: Experimental human-in-the-loop study. Wearable Technologies 4, e13. 38487766 10.1017/wtc.2023.9 PMC10936398

[r44] NIOSH (2019) *Niosh Strategic Plan: Fys 2019–2023.*

[r45] Otten BM, Weidner R and Argubi-Wollesen A (2018) Evaluation of a novel active exoskeleton for tasks at or above head level. IEEE Robotics and Automation Letters 3(3), 2408–2415.

[r46] Pacifico I, Scano A, Guanziroli E, Moise M, Morelli L, Chiavenna A, Romo D, Spada S, Colombina G, Molteni F, et al (2020) An experimental evaluation of the proto-mate: A novel ergonomic upper-limb exoskeleton to reduce workers’ physical strain. IEEE Robotics & Automation Magazine 27(1), 54–65.

[r47] Park D, Toxiri S, Chini G, Di Natali C, Caldwell DG and Ortiz J (2021) Shoulder-sidewinder (shoulder-side wearable industrial ergonomic robot): Design and evaluation of shoulder wearable robot with mechanisms to compensate for joint misalignment. IEEE Transactions on Robotics. 38(3), 1460–1471.

[r48] Pedrocchi N, Vicentini F., Matteo M. and Tosatti LM (2013) Safe human-robot cooperation in an industrial environment. International Journal of Advanced Robotic Systems 10(1), 27.

[r49] Radzevich SP (2016) Dudley’s Handbook of Practical Gear Design and Manufacture. CRC Press.

[r50] Ramella G, Grazi L, Giovacchini F, Trigili E, Vitiello N and Crea S (2024) Evaluation of antigravitational support levels provided by a passive upper-limb occupational exoskeleton in repetitive arm movements. Applied Ergonomics 117, 104226. 38219374 10.1016/j.apergo.2024.104226

[r51] Rodriguez-Cianca D, Rodriguez-Guerrero C, Verstraten T, Jimenez-Fabian R, Vanderborght B and Lefeber D (2019) A flexible shaft-driven remote and torsionally compliant actuator (rtca) for wearable robots. Mechatronics 59, 178–188.

[r52] Rossini M, De Bock S, Ducastel V, Langlois K, De Pauw K, Geeroms J … and Lefeber D (2022) Effectiveness of a passive neck support mechanism for overhead occupational tasks. In 2022 9th IEEE RAS/EMBS International Conference for Biomedical Robotics and Biomechatronics (BioRob), 1–6. IEEE.

[r53] Rossini M, De Bock S, van der Have A, Flynn L, Rodriguez-Cianca D, De Pauw K, … and Rodriguez-Guerrero C (2021) Design and evaluation of a passive cable-driven occupational shoulder exoskeleton. IEEE Transactions on Medical Robotics and Bionics 3(4), 1020–1031.

[r54] Rossini M., Geeroms J., Lefeber D. and Rodriguez-Guerrero C (2021c) Automatic synthesis of arthrokinematically compatible exoskeletons. A case study on its application on a shoulder occupational exoskeleton. Mechanism and Machine Theory, 157, 104186.

[r55] Rudnicka E, Napierala P, Podfigurna A, Meczekalski B, Smolarczyk R and Grymowicz M (2020) The world health organization (who) approach to healthy ageing. Maturitas 139, 6–11. 32747042 10.1016/j.maturitas.2020.05.018 PMC7250103

[r56] Sänger J, Yao Z., Schubert T, Wolf A, Molz C, Miehling J, Wartzack S, Gwosch T, Matthiesen S and Weidner R (2022) Evaluation of active shoulder exoskeleton support to deduce application-oriented optimization potentials for overhead work. Applied Sciences 12(21), 10805.

[r57] Schiele A (2009) Ergonomics of exoskeletons: Objective performance metrics. In World Haptics 2009 - Third Joint EuroHaptics conference and Symposium on Haptic Interfaces for Virtual Environment and Teleoperator Systems, 103–108. IEEE. 10.1109/WHC.2009.4810871

[r58] Sylla N, Bonnet V, Colledani F and Fraisse P (2014) Ergonomic contribution of able exoskeleton in automotive industry. International Journal of Industrial Ergonomics, 44(4), 475–481.

[r59] Toxiri S, Näf MB, Lazzaroni M, Fernández J, Sposito M, Poliero T, Monica L, Anastasi S, Caldwell DG and Ortiz J (2019) Back-support exoskeletons for occupational use: An overview of technological advances and trends. IISE Transactions on Occupational Ergonomics and Human Factors, 7(3–4), 237–249.

[r60] van der Have A, Rossini M, VanRossom S and Jonkers I (2021) The exo4work shoulder exoskeleton effectively reduces muscle and joint loading during occupational tasks above shoulder height. Applied Ergonomics *Under Revision* (1), 22–122. 10.1016/j.apergo.2022.103800 35598416

[r61] Van Engelhoven L., Poon N., Kazerooni H., Rempel D., Barr A. and Harris-Adamson C (2019) Experimental evaluation of a shoulder-support exoskeleton for overhead work: Influences of peak torque amplitude, task, and tool mass. IISE Transactions on Occupational Ergonomics and Human Factors 7(3–4), 250–263.

[r62] Verhagen AP, Bierma-Zeinstra SM., Burdorf A, Stynes SM, deVet HC, and Koes BW (2013) Conservative interventions for treating work-related complaints of the arm, neck or shoulder in adults. Cochrane Database of Systematic Reviews (12). 10.1002/14651858.CD008742.pub2 PMC6485977 24338903

[r63] Verstraten T, Beckerle P, Furnémont R, Mathijssen G, Vanderborght B and Lefeber D (2016) Series and parallel elastic actuation: Impact of natural dynamics on power and energy consumption. Mechanism and Machine Theory 102, 232–246.

[r64] Ye W and Li Q (2019) Type synthesis of lower mobility parallel mechanisms: A review. Chinese Journal of Mechanical Engineering 32(1), 38.

